# A Qualitative Analysis of Malpractice Litigation in Cardiology Using Case Summaries Through a National Legal Database Analysis

**DOI:** 10.7759/cureus.5259

**Published:** 2019-07-28

**Authors:** Richa Patel, Nicole Rynecki, Eric Eidelman, Spandana Maddukuri, Varun Ayyaswami, Manthan Patel, Raghav Gupta, Arpan V Prabhu, Jared Magnani

**Affiliations:** 1 Medicine, Rutgers University New Jersey Medical School, Newark, USA; 2 Medicine, University of Maryland School of Medicine, Baltimore, USA; 3 Medicine, Philadelphia College of Osteopathic Medicine, Philadelphia, USA; 4 Radiation Oncology, Winthrop P. Rockefeller Cancer Institute, University of Arkansas for Medical Sciences, Little Rock, USA; 5 Cardiology, University of Pittsburgh Medical Center Heart and Vascular Institute, Pittsburgh, USA

**Keywords:** medical malpractice, cardiology, legal database analysis

## Abstract

Introduction

Physicians are increasingly practicing defensive medicine as a response to society’s litigious climate. This study sought to characterize cardiology malpractice claims and elucidate the allegations underlying the use of defensive medicine.

Methods

The WestlawNext™ database was queried to obtain state and federal jury verdicts and settlements related to medical malpractice and cardiology that occurred in the United States between 2010 and 2015. Cardiology cases were identified using the search terms “medical malpractice” and “cardiology” and reviewed by two individuals utilizing available case documents. Duplicate and nonpertinent cases were excluded. Binary logistic regression models were created to predict the likelihood of defendant verdict, plaintiff verdict, and settlement based on the various reasons for litigation cited.

Results

Inclusion criteria were met in 166 cases. The plaintiffs were predominantly male (94 cases; 56.6%), and the average patient age was 53.3±17.5 years. More than half of the cases involved a cardiologist as a defendant. The most common reasons for litigation were: failure to treat (129; 77.7%), failure to diagnose (115; 69.3%), failure to refer/order diagnostic tests (107; 64.5%), and patient death (118; 71.1%). Among cases tried for failure to diagnose, the most commonly missed diagnosis was myocardial infarction. Cases most commonly resulted in a defendant verdict (94; 56.6%). However, odds of a plaintiff verdict were significantly higher when failure to diagnose was alleged with an odds ratio (OR) of 7.60 (95% confidence interval 1.14 - 50.87, p = 0.0365).

Conclusions

Failure to diagnose remains a commonly alleged base for litigation. In conclusion, our analysis suggests increased training for non-cardiologists in the recognition of the acute coronary syndrome and enhanced awareness of inherent biases among all physicians may facilitate reducing missed diagnoses.

## Introduction

Physicians’ principal duty is to provide the highest quality medical care for patients. However, fear of litigation may impact how physicians approach and evaluate potential diagnoses. The use of defensive medicine, or medical practices performed to protect physicians from liability claims, has become the norm in many contemporary medical practices [1-3]. Physicians may order diagnostic imaging or procedures to rule out serious and even unlikely diagnoses to reduce the possibility of litigation [2]. Defensive medicine has been criticized because of its contribution towards increased healthcare costs; it is estimated that the costs of defensive medicine totaled over $45 billion dollars in 2008 [4]. US cardiologists are more likely to face malpractice claims than non-cardiologists (8.6% versus 7.4%), and litigation rates for cardiologists are surpassed only by gastroenterologists and cardiothoracic surgeons [5]. Previous characterization of cardiology medical malpractice claims found that diagnostic error was the leading cause for litigation [5, 6].

We queried WestlawNext™, an online database of legal proceedings, to characterize cardiology malpractice litigation from 2010 to 2015. Our goals were twofold: first, we sought to characterize the reasons for litigation of medical malpractice claims against defendant cardiologists; second, we aimed to determine the associations between the cited bases for litigation and defendant or plaintiff verdicts. Previous literature has indicated that the tendency to litigate is largely based on the patient’s perception of the doctor-patient relationship [7, 8]. As a result, we hypothesize that despite a climate of increased use of defensive medicine, failure to diagnose remains a common reason for litigation among cardiology cases and is associated with increased odds of plaintiff verdict.

## Materials and methods

The WestlawNext™ legal database (Thomson Reuters, New York, NY) was used to characterize cardiology malpractice-related state and federal jury verdict and settlement reports in the United States between January 1, 2010 and December 31, 2015 [9]. The WestlawNext™ registry is widely used for legal research and incorporates multiple legal databases with an advanced search algorithm [10]. Results include relevant case documents such as jury verdicts, settlements, and case summaries, thereby allowing for review of litigated cases. The database is updated regularly with content supervised by attorney editors responsible for accurately categorizing and summarizing cases, and has had extensive application in analyses of medical malpractice [11-13].

We queried WestlawNext™ with the search terms “medical malpractice” and “cardiology.” Two independent reviewers (Varun Ayyaswami - VA and Eric Eidelman - EE) reviewed the available documents for the 224 cases identified by the search. The reviewers examined 10 cases to ensure consistent categorization followed by each reviewer examining 50% of the remaining cases. Reviewers identified duplicate cases and those not pertinent to cardiovascular disease during review. The following were extracted from the remaining cases for each verdict or settlement: the state in which the trial was conducted, defendant specialty and membership in hospital group, plaintiff age and sex, reasons for litigation, patient death, jury verdict, and value of monetary award. The reasons for litigation were determined for each case through review of available case files and cases were classified with one or more reasons for litigation as used by previous malpractice studies [9, 11, 14]. Reasons for litigation were characterized by our reviewers as failure to treat, failure to diagnose, failure to refer/order diagnostic tests, procedural error, severe hospitalization, unnecessary surgery, lack of informed consent, and death.

Binary logistic regression models were created to predict likelihood of defendant verdict versus plaintiff verdict, defendant verdict versus case settlement, and plaintiff verdict versus case settlement. Odds ratios (OR) and 95% confidence intervals (CI) were calculated for patient age greater than 65 years and each reason for litigation. A p­-value of <0.05 was considered statistically significant. Regressions were performed in SAS Enterprise Guide 7.1 (SAS Institute Inc., Cary, NC, USA).

## Results

**Figure 1:**
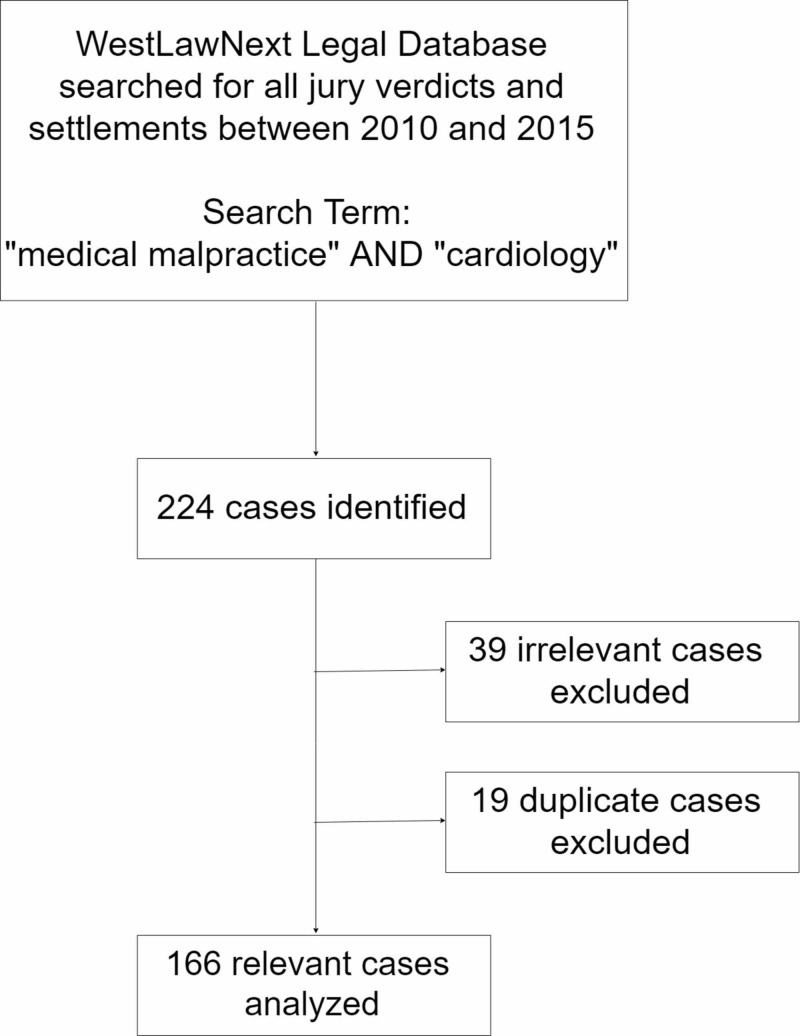
Isolation of cases

Following exclusions for duplicate cases (n=16) or not related to cardiovascular disease (n=39), there were 166 cases available for review, as summarized by Figure 1. Plaintiffs for the 166 cases were predominantly male (n=94; 56.6%), and the age of the affected patient was 53.3±17.5 years. Table 1 summarizes characteristics of the cases and Table 2 presents reasons for litigation. Cases were distributed amongst 24 US states with the majority being tried in Florida (30; 18.1%) followed by New York (19; 11.5%), California, and Massachusetts (both 18; 10.8%) (Table 3).

**Table 1:** Malpractice litigation related to cardiology 2010-2015

Patient Demographics	
Number of total cases	224
Number of irrelevant cases	39
Number of duplicate cases	19
Number of studied cases	166
Gender of plaintiff	
Male	94 (56.6%)
Female	68 (40.9%)
Total unknown (not available or confidential)	4 (2.4%)
Mean age (years; range)	52.48; (2 months – 89 years)
Cases with just an age range (no specific age)	9 (5.4%)
Total unknown age (not available or confidential)	39 (23.5%)
Number of defendants	
1	58 (34.9%)
2	51 (30.7%)
3	22 (13.3%)
4	9 (5.4%)
5	7 (4.2%)
6	9 (5.4%)
7	3 (1.8%)
9	3 (1.8%)
10	1 (0.6%)
Not available or confidential	3 (1.8%)
Cases that involved a cardiologist as a defendant	109 (65.7%)
Hospital or medical group involvement	
Yes	107 (64.9%)
No	55 (33.3%)
Unknown	3 (1.8%)
Year verdict rendered	
2010	51 (30.7%)
2011	31 (18.7%)
2012	23 (13.9%)
2013	27 (16.2%)
2014	10 (6%)
2015	22 (13.3%)
Not available or confidential	2 (1.2%)
Jury verdict	
Defendant	94 (56.6%)
Plaintiff	40 (24.1%)
Settlement	30 (18.1%)
Mixed	1 (0.6%)
Other	1 (0.6%)
Mean payouts; (range)	$2,266,745.503; ($20,000.00 - $126,642,039.00)
Mean plaintiff verdict payout (cases, range)	$7,213,287.82 (39, $325,000 – $126,642,039)
Mean settlement verdict payout (cases, range)	$2,648,881.44 (27, $100,000 – $17,000,000)
Mixed verdict payout (one case)	$551,500

**Table 2:** Reasons for litigation

Reason for Litigation	Total Cases: 166
Failure to treat	129 (77.7%)
Failure to diagnose	115 (69.3%)
Failure to refer/order diagnostic tests	107 (64.5%)
Other	43 (26%)
Procedural error	32 (19.2%)
Severe hospitalization	7 (4.2%)
Unnecessary surgery	4 (2.4%)
Lack of informed consent	2 (1.2%)
Death	118 (71.1%)
One error	7 (4.21%)
Two errors	30 (18.1%)
Three errors	42 (25.3%)
Four errors	72 (43.4%)
Five errors	13 (7.8%)
Six errors	1 (0.6%)

**Table 3:** Geographic distribution of malpractice cases

Geographic Distribution	
Florida	30 (18.1%)
New York	19 (11.5%)
California	18 (10.8%)
Massachusetts	18 (10.8%)
Pennsylvania	14 (8.4%)
Illinois	11 (6.6%)
Texas	7 (4.2%)
Connecticut	6 (3.6%)
Michigan	6 (3.6%)
New Jersey	5 (3%)
Ohio	5 (3%)
Washington	5 (3%)
Alabama	4 (2.4%)
Missouri	4 (2.4%)
Delaware	2 (1.2%)
Indiana	2 (1.2%)
New Hampshire	2 (1.2%)
Oklahoma	2 (1.2%)
Arizona	1 (0.6%)
Kansas	1 (0.6%)
Louisiana	1 (0.6%)
Minnesota	1 (0.6%)
Montana	1 (0.6%)
Virginia	1 (0.6%)

The most common reasons for litigation were failure to treat (129; 77.7%), death (118; 71.1%), failure to diagnose (115; 69.3%), and failure to refer/order diagnostic tests (107; 64.5%). Among cases involving failure to diagnose as a reason for litigation, the most commonly missed diagnosis was “myocardial infarction”, which occurred in 32 cases (19.3%) (Table 4). Other frequently missed diagnoses were coronary artery disease, aortic dissection, pulmonary embolism, congestive heart failure, and stroke. Missed referrals included surgical and cardiology referrals, whereas missed diagnostic tests included cardiac catheterizations, electrocardiograms, and cardiac biomarkers. The lack of informed consent category under reasons for litigation was excluded from analysis as only two cases involved informed consent, both of which resulted in defendant verdicts.

**Table 4:** Most commonly missed diagnoses in cases involving “failure to diagnose” as reason for litigation

Diagnosis	Number of Cases
Myocardial infarction	32 (19.3%)
Coronary artery disease	6 (3.6%)
Aortic dissection	5 (3.0%)
Pulmonary embolism	5 (3.0%)
Congestive heart failure	4 (2.4%)
Stroke	4 (2.4%)
Arterial occlusion of femoral artery	3 (1.8%)
Coronary artery dissection	3 (1.8%)
Arrythmia	2 (1.2%)
Cardiovascular disease	2 (1.2%)
Endocarditis	2 (1.2%)
Heart disease	2 (1.2%)
Hypertrophic obstructive cardiomyopathy	2 (1.2%)
Pericarditis	2 (1.2%)

The number of defendants per case spanned from one to 10, with the majority involving either one (58, 34.9%) or two defendants (51, 30.7%). About two thirds of cases involved a cardiologist as a defendant (109; 65.6%) and a similar proportion involved a hospital or medical group (107, 64.9%).

Verdicts were in favor of the defendant in 94 cases (56.6%). Plaintiff verdicts and settlements were reached in 24.1% and 18.1% of cases, respectively. Two cases (1.2%) had mixed or uncategorized outcomes. The average payout overall was $2,266,745.50 with a range of $20,000.00 - $126,642,039.00.

Outcomes from regression analysis are presented in Table 5 and indicate that failure to diagnose had significantly higher odds of plaintiff verdict compared to defendant verdict (OR 7.60 (1.14, 50.87), p = 0.04). Patient age ≥65 years, failure to treat in a timely manner, failure to refer/order diagnostic tests, unnecessary surgery, procedural error, severe hospitalization greater than 30 days, and patient death were not associated with jury or settlement outcomes.

**Table 5:** Logistic regression analysis

	Odds of defendant verdict versus plaintiff verdict		Odds of defendant verdict versus settlement		Odds of plaintiff verdict versus defendant verdict		Odds of plaintiff verdict versus settlement	
Covariate	OR (95% CI)	p value	OR (95% CI)	p value	OR (95% CI)	p value	OR (95% CI)	p value
Patient age greater or equal to 65 years old	1.84 (0.60, 5.71)	0.2885	4.18 (0.73, 23.81)	0.1074	0.54 (0.18, 1.68)	0.2885	2.27 (0.35, 14.81)	0.3934
Failure to diagnose	0.13 (0.02, 0.88)	0.0365	0.65 (0.13, 3.19)	0.5927	7.60 (1.14, 50.87)	0.0365	4.92 (0.51, 47.26)	0.1677
Failure to treat in a timely manner	2.66 (0.35, 20.26)	0.3461	2.580 (0.34, 19.46)	0.3578	0.38 (0.05, 2.87)	0.3461	0.9722 (0.08, 12.27)	0.9822
Failure to refer/order diagnostic tests	1.57 (0.45, 5.49)	0.4813	0.241 (0.04, 1.61)	0.1418	0.64 (0.18, 2.23)	0.4813	0.15 (0.02, 1.26)	0.0810
Unnecessary surgery			0.222 (0.01, 3.96)	0.3061				
Procedural error	2.23 (0.53, 9.49)	0.2771	0.506 (0.09, 2.71)	0.4268	0.45 (0.11, 1.91)	0.2771	0.23 (0.03, 1.70)	0.1491
Severe hospitalization	0.86 (0.09, 8.38)	0.8981	0.268 (0.03, 2.50)	0.2483	1.16 (0.12, 11.28)	0.8981	0.31 (0.03, 3.22)	0.3278
Death	1.34 (0.534 3.35)	0.5318	0.57 (0.17, 1.93)	0.3615	0.75 (0.30, 1.87)	0.532	0.42 (0.11, 1.64)	0.2123

## Discussion

The primary goal of this study was to characterize reasons for litigation against cardiologists and to determine if they have remained static in the setting of a changing medicolegal landscape. Our analysis reveals that despite increased use of defensive medicine in recent years, failure to diagnose remained among failure to treat and patient death as the most commonly cited bases for litigation. Among cases that cited failure to diagnose as a reason for litigation, myocardial infarction was the most commonly missed diagnosis.

It is paradoxical that even with documented increases in defensive medicine, physicians are still frequently facing malpractice claims that cite missed diagnoses [4, 15, 16]. This study is unique in that in addition to characterizing cardiology claims, it provides a framework for understanding the predictors of a plaintiff verdict. Therefore, we were able to demonstrate that in addition to the sheer frequency of cases involving failure to diagnose, claims with this basis for litigation cited have a statistically greater odds of a plaintiff verdict. The association of failure to diagnose with a plaintiff verdict is consistent with analyses of litigation in other medical specialties. In a study of otolaryngology malpractice litigation, failure to diagnose was the most common legal allegation, encompassing 51.5% of cases [17]. In a report on neurosurgery malpractice, failure to diagnose was listed as the third most common reason for litigation preceded only by procedural error and failure to treat [18]. It is critical that future research investigates the systematic reasons as to why diagnoses are missed.

Our study is not the first to ascertain that missed diagnoses of myocardial infarction are at the crux of much of cardiology malpractice litigation [19]. A 2017 retrospective analysis of malpractice claims involving myocardial infarction found that misdiagnosis was the most common claim. The analysis also found that delayed diagnosis resulted in a plaintiff verdict if the physician either failed to work up a patient with coronary artery disease risk factors presenting with the cardinal symptom of chest pain or ischemic heart disease or if the physician failed to perform indicated treatment in a timely manner to avoid disease progression [20]. Of note, cited bases for litigation are obtained from legal proceedings, and the semantics used in law may differ from those in medicine. In the setting of an acute myocardial event, missed diagnosis, misdiagnosis, and delayed diagnosis often have similar morbidity and patient outcomes. We, therefore, argue that the finding of delayed diagnosis resulting in a plaintiff verdict corroborates our finding of failure to diagnose resulting in a plaintiff verdict.

Defensive medicine is often critiqued for subjecting patients to a greater number of tests and increasing healthcare costs, and current literature remains inconclusive as to whether defensive medicine has patient merit. In an analysis of patients with acute myocardial infarctions admitted to California hospitals, patients in the highest quintile of hospital spending had lower inpatient mortality rates compared to those in the lowest quintile [21]. Increased hospital spending correlated with a greater number of diagnostic tests ordered. Another study amongst internists found that the internists in the highest fifth of patient risk-adjusted resource use were approximately half as likely to face future malpractice claims when compared to internists in the lowest fifth [22]. Increased use of resources was associated with both a decrease in litigation and improved patient health outcomes. Contrary to the findings of these two studies, a recent study of defensive medicine in the military, a setting in which physicians are immune from medical malpractice lawsuits, indicated that liability immunity reduced inpatient spending by 5%, with no negative ramifications on patient outcomes [23]. Frakes et al. argued that because defensive medicine is conducted unsystematically and without proper clinical suspicion, it does not result in improvements in patient care [23]. Furthermore, eliminating the impetus for defensive medicine via medical malpractice immunity did not decrease the quality of patient care [23, 24].

We also identified that cardiologists were not defendants in a large portion of cases. The high percentage of non-cardiologists implicated in cardiology medical malpractice claims highlights the role of other specialties and healthcare professionals in recognizing and managing acute cardiac events. Due to the acuity of myocardial infarctions, patients may not necessarily present to a cardiologist, often presenting to a primary care physician or the emergency department. It can be argued that in this regard, ordering an electrocardiogram may be an appropriate constitution of defensive medicine, as the consequences of a missed diagnosis of myocardial infarction are detrimental, and this inexpensive test likely does not constitute a significant portion of defensive medicine’s contribution to rising healthcare costs. However, the failure of this method in detecting myocardial infarctions lies in the interpretations of the electrocardiograms. Studies have found that despite the introduction of computerized interpretations, medical students and physicians of a variety of specialties may not be adequately trained to read electrocardiograms, and this can lead to adverse outcomes [25].

Increased education in cultural competencies and atypical presentations of myocardial infarction may also play a pivotal role in decreasing missed diagnoses of myocardial infarction. Women less than the age of 55 and non-white individuals presenting to the emergency room with symptoms of acute cardiac ischemia are significantly less likely to be hospitalized than men and white counterparts, respectively [26]. Inherit biases may also play a role in missing diagnoses. Diagnostic errors have previously been categorized as either no-fault errors, system-related errors, cognitive-related errors, or a mixture of all three [27]. Cognitive related errors are, to some degree, a result of inherent biases and have been shown to contribute to physicians’ assessments of patients’ presenting symptoms. Therefore, missed diagnoses may be reduced by training physicians to be more cognizant of subconscious biases during patient assessments. 

There are several limitations to this study. Although the WestlawNext™ search engine includes large numbers of cases from federal and state courts, case submission is not mandatory, and the database does not contain out of court malpractice settlements. As a result, our sample size was limited. For example, the initial review of the 166 cases that met inclusion criteria revealed too few cases involving unnecessary surgery and failure of informed consent as reasons for litigation to calculate odds ratios for verdict outcomes. It is possible that such allegations have limited incidence in cardiology. For example, while there are certainly variations in the procurement of informed consent, physicians across all specialties are aware of the legal consequences of performing procedures without consent, more so than ever before [28]. Additionally, defendant physician professional and demographic information such as years of experience, age, history of previous litigation, and medical education were not consistently available for analysis. Decreased age, previous litigation, and lower quality medical education based on national rankings of institutions are all independently associated with increased odds of being involved in malpractice suits [29]; we were unable to investigate the associations of these factors in our analysis.

## Conclusions

This analysis of cardiology malpractice claims over a six-year period confirmed our hypothesis that failure to diagnose is a common reason for litigation and a statistically significant predictor of a plaintiff verdict; this is despite documented increases in defensive medicine over the past decade. While this study cannot isolate the impact of defensive medicine on missed cardiovascular diagnoses, it is imperative to recognize that defensive medicine alone is evidently insufficient to protect both physicians and patients. As myocardial infarction was the most frequently missed diagnosis, further research is warranted to investigate downfalls in diagnosis with a focus on electrocardiogram interpretation and cultural competency. Attention to this diagnosis has the potential to improve patient outcomes, decrease the burden of medical malpractice claims, and optimize the allocation of healthcare dollars.
